# Piloting co-developed behaviour change interventions to reduce exposure to air pollution and improve self-reported asthma-related health

**DOI:** 10.1038/s41370-024-00661-2

**Published:** 2024-04-12

**Authors:** Amy McCarron, Sean Semple, Vivien Swanson, Colin Gillespie, Christine Braban, Heather D. Price

**Affiliations:** 1 Biological and Environmental Sciences, University of Stirling, Stirling, UK; 2 Institute of Social Marketing and Health, University of Stirling, Stirling, UK; 3 Psychology, University of Stirling, Stirling, UK; 4 Scottish Environment Protection Agency (SEPA), Stirling, UK; 5 UK Centre for Ecology and Hydrology (UKCEH), Penicuik, UK

**Keywords:** Personal exposure, Particulate Matter, Health studies

## Abstract

**Background:**

Exposure to air pollution can exacerbate asthma with immediate and long-term health consequences. Behaviour changes can reduce exposure to air pollution, yet its ‘invisible’ nature often leaves individuals unaware of their exposure, complicating the identification of appropriate behaviour modifications. Moreover, making health behaviour changes can be challenging, necessitating additional support from healthcare professionals.

**Objective:**

This pilot study used personal exposure monitoring, data feedback, and co-developed behaviour change interventions with individuals with asthma, with the goal of reducing personal exposure to PM_2.5_ and subsequently improving asthma-related health.

**Methods:**

Twenty-eight participants conducted baseline exposure monitoring for one-week, simultaneously keeping asthma symptom and medication diaries (previously published in McCarron et al., 2023). Participants were then randomised into control (*n* = 8) or intervention (*n* = 9) groups. Intervention participants received PM_2.5_ exposure feedback and worked with researchers to co-develop behaviour change interventions based on a health behaviour change programme which they implemented during the follow-up monitoring week. Control group participants received no feedback or intervention during the study.

**Results:**

All interventions focused on the home environment. Intervention group participants reduced their at-home exposure by an average of 5.7 µg/m³ over the monitoring week (−23.0 to +3.2 µg/m³), whereas the control group had a reduction of 4.7 µg/m³ (−15.6 to +0.4 µg/m³). Furthermore, intervention group participants experienced a 4.6% decrease in participant-hours with reported asthma symptoms, while the control group saw a 0.5% increase. Similarly, the intervention group’s asthma-related quality of life improved compared to the control group.

**Impact statement:**

This pilot study investigated a novel behaviour change intervention, utilising personal exposure monitoring, data feedback, and co-developed interventions guided by a health behaviour change programme. The study aimed to reduce personal exposure to fine particulate matter (PM_2.5_) and improve self-reported asthma-related health. Conducting a randomised controlled trial with 28 participants, co-developed intervention successfully targeted exposure peaks within participants’ home microenvironments, resulting in a reduction in at-home personal exposure to PM_2.5_ and improving self-reported asthma-related health. The study contributes valuable insights into the environmental exposure-health relationship and highlights the potential of the intervention for individual-level decision-making to protect human health.

## Introduction

Exposure to air pollution poses a significant public health threat and, globally, is responsible for 7 million premature deaths every year [1] owing to illnesses such as asthma, chronic obstructive pulmonary disease (COPD) and lung cancer [2]. The health impacts of air pollution span the entire life course, with foetal exposure resulting in adverse birth outcomes such as low birth weight and pre-term birth; childhood and adolescent exposure linked with, among others, physical and psychological developmental issues; and exposure in adulthood and old age associated with cardiovascular and respiratory ill-health and premature death [3]. Additionally, air pollution is a known trigger which can exacerbate existing illnesses and has been associated with both acute asthma exacerbations and the longer-term deterioration of the condition [4]. Fine particulate matter is a key pollutant from a respiratory health perspective since it can be deposited throughout the respiratory tract, particularly in small airways and alveoli [5]. As such, people with pre-existing respiratory conditions such as asthma, COPD or bronchiectasis, are considered a ‘vulnerable’ group for whom exposure to air pollution should be minimised [6].

Air quality-related policies tend to focus on emission reductions rather than exposure prevention [7]. While they can be effective for improving ambient air quality, they are slow to implement and even slower to produce tangible effects [8]. Additionally, as they are designed to benefit entire communities, a policy approach tends to be a broad brush, one-size-fits-all approach (e.g., low emission zones), and does not provide those most vulnerable with targeted solutions to reduce their personal vulnerability. It has been argued that individual behaviours and behavioural patterns can have a more significant role in influencing personal exposure than ambient pollution levels [9]. Further, such behavioural changes can be easier to implement, can give people autonomy over their personal exposures, and can have a more immediate health impact [10] (though the burden of responsibility should not solely be with the individual [11]). Behaviour changes can also be better targeted for the individual, recognising the nuances in personal exposure and allowing individuals to take protective and proactive control over their exposure-related health. Behavioural changes are therefore potentially very beneficial for supporting the non-pharmacological self-management of pre-existing respiratory conditions such as asthma [12, 13]. Individual-level behaviour change, alongside policy changes, could therefore have a key role to play in reducing the health impacts associated with exposure to air pollution [14], particularly for vulnerable groups [7].

Resources aimed at encouraging individual-level behaviour change (e.g., the UK’s Daily Air Quality Index (DAQI) and the U.S.’s Air Quality Activity Guide) recommend exposure minimising behaviours such as reducing or avoiding outdoor activities. However, these resources, focusing on avoidance and reduction behaviours, do not empower change and are therefore unlikely to significantly impact behaviour change owing to the lack of personalisation of air quality data and lack of individual participation in developing feasible behaviour changes [10]. Moreover, engagement with such resources tends to be stratified, with some groups of people more likely to access these data and information than others and interaction does not necessarily translate into action [15]. Howard [16] and others have called for information on the health impacts of air pollution to become more integrated into clinical practice, yet how this is implemented in a way that both personalises the air quality information and engages individuals in developing behaviour changes is still to be investigated. Progress is being made in this regard. For example, a recent initiative in London, UK led by Great Ormond Street Hospital and Imperial College London reports annual average pollution levels for patients’ postcodes on their medical records as a way of ‘personalising’ the risk of air pollution and initiating conversations [17]. However, this falls short of providing practical, personalised advice as to how to reduce personal exposures via behavioural modifications.

Accessing more personalised air quality data can motivate protective health behaviours by targeting an individual’s threat appraisal (how one perceives the threat of air pollution to their own health) and coping appraisal (how one perceives their ability to overcome the threat of air pollution) [10, 18]. However, motivation alone is insufficient to initiate behaviour change [19]. Instead, it represents the initial stage of a multi-step process [10]. Recognising that individuals engage in two distinct types of cognitive processes when making decisions—reflective processes that involve deliberate and conscious thinking, and automatic processes that operate intuitively and unconsciously—the next step, moving beyond motivation and initiating action, requires the development of action and coping plans [20]. Action planning involves developing a specific and detailed plan outlining the steps the individual will take to initiate a health-related behaviour change, detailing, for example, when and where the behaviour change will take place (for example as a hypothetical illustration, “I will open a window when I am frying food in the kitchen”). Coping planning focuses on overcoming barriers to initiating or maintaining the behaviour change by identifying potential setbacks and planning solutions. For example, “I will leave a jumper in the kitchen so that if it is too cold with the window open, I can put it on”. Reflective processes play a crucial role in shaping these plans, as it requires the conscious assessment of perceived benefits of these actions. Additionally, integrating automatic processes through environmental cues and habit formation can further reinforce health behaviour change. For instance, incorporating a visual cue, such as placing an air quality monitor in a prominent location, or establishing a daily routine for checking air quality data, can contribute to the integration of health behaviour changes. This intertwining of reflective and automatic processes enhances the likelihood of sustained health behaviour change. Health behaviour change can be challenging, but the process can be facilitated with help and support from a healthcare professional [21].

The ‘MAP (Motivation, Action and Prompts) of health behaviour change’ [22] is a tool developed by the National Health Service (NHS) in Scotland, UK, to guide individual behaviour change practice for improved health [23]. The function of MAP as a behaviour change support tool is to aid health and care staff to support service users to make sustainable behaviour changes to positively influence their physical health, mental health and general wellbeing. This recognises that for a sustainable behaviour change to occur, individuals must be **m**otivated to make the change, take **a**ction to alter their behaviour(s) and have awareness of the **p**rompts and cues which can both support and hinder the behaviour change. It provides a simple and accessible, yet theoretically informed guide to identify the most appropriate behaviour change techniques to employ to achieve the desired outcome. Most closely, the ‘MAP of health behaviour change’ draws on Schwarzer’s [20] Health Action Process Approach (HAPA) model of self-regulatory behaviour change focusing on pre-intentional and intentional (enactment) phases. It also incorporates ‘dual process’ models of thinking, targeting both reflective and automatic routes. Yet, a critical benefit of the ‘MAP of health behaviour change’ is its accessibility to the non-specialist user (e.g., asthma nurses) while being theoretically situated, without requiring input from behavioural scientists which would command significant time and resource for intervention development [24]. Therefore, it could be an efficient and effective tool to develop tailored behaviour changes for personal exposure reduction. To date, to the authors’ knowledge, the ‘MAP of health behaviour change’ has only been applied to the typical priorities of the NHS in Scotland, such as to provide support for smoking cessation or exercise uptake behaviour change [22].

This study therefore had two main aims. The first, to test the method of using wearable sensors for personal exposure monitoring, data feedback, and co-developing behaviour change interventions structured around the ‘MAP of health behaviours change’. The second aim was to assess its efficacy in reducing personal PM_2.5_ exposure, with the hypothesis that this may subsequently improve self-reported asthma-related health.

## Methods

### Study design and participants

**Fig. 1:**
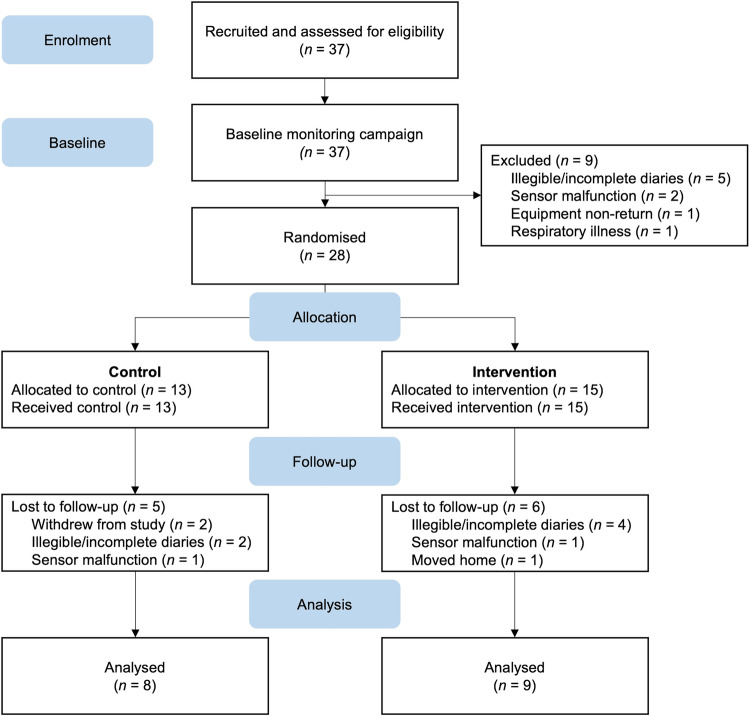
CONSORT-style flow diagram illustrating the flow of participants from recruitment through baseline and follow-up campaigns. Results from the baseline campaign are published in McCarron et al. [26].

Between February 2021 and July 2021, 37 participants were recruited from across Scotland to take part in the study. To be eligible to participate, participants had to have received an asthma diagnosis from a healthcare professional, be aged 18 or older, be a non-smoker, and live in Scotland. Participants were enroled as part of a larger study in which they were interviewed about their lived experience of asthma in relation to air pollution [25], before measuring their personal exposure to air pollution (hereafter called the baseline campaign) [26], and then taking part in the study presented here. Overall, each participant took part in the study for (approximately) one month. A participant advisory group comprised of five individuals meeting the same eligibility criteria helped refine the project design and test the methodology during a pre-pilot phase (detailed in [26]). Data collection took place between September 2021 and September 2022 following a parallel group randomised control trial design. All participants who conducted baseline monitoring were allocated at random to either the control or intervention study arm before conducting follow-up monitoring (Fig. 1). Ethical approval for this study was provided by the University of Stirling’s General University Ethics Panel [GUEP 2021 2506 1892].

### Personal exposure monitoring and self-reported asthma-related health

Full details of the personal exposure monitoring methodology and baseline campaign are detailed in [26] and summarised here.

**Fig. 2:**
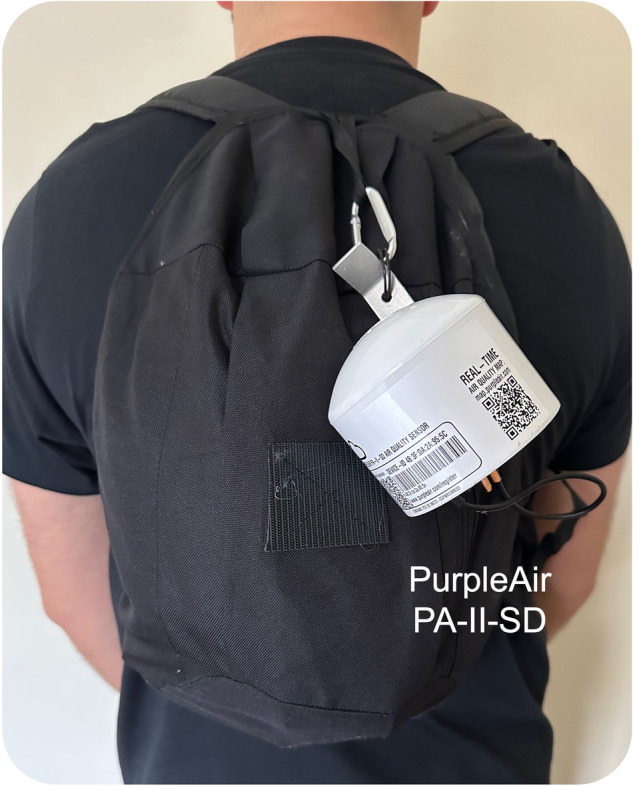
PurpleAir attached to customised backpack and powered by battery pack (inside). The PurpleAir was secured in place with Velcro to minimise agitating fibre particles and to keep the sensor as close as feasibly possible to ‘breathing zone’ height. When stationary for long periods, the participant was permitted to remove the PurpleAir from the backpack and keep it close-by (as in McCarron et al. [26]).

Personal exposure to fine particulate matter (particulate matter with an aerodynamic diameter $\le \!\!2.5$ μm (PM_2.5_)), was individually monitored by each participant using a custom-designed backpack carrying a PurpleAir PA-II-SD air quality sensor (hereafter referred to as PurpleAir) (Fig. 2). To capture participants’ weekly routines and typical weekly variations in ambient PM_2.5_ concentrations, data collection took place over one week at baseline and, approximately 1-month later, over one week at follow-up. The PurpleAir uses Plantower PMS 5003 air quality sensors in addition to measuring relative humidity, temperature, and barometric pressure (Bosch, Reutlingen, Germany). Laser counters record readings every five seconds, with 120-s averages stored on an SD card.

Before data collection commenced, all 16 PurpleAir devices used in this study were co-located for one week to ensure inter-unit comparability [27]. Given that co-location with a reference-grade monitor was not possible owing to fieldwork restrictions during the COVID-19 pandemic, the median value across all 16 sensors was accepted as the ‘true’ value [28]. Individual sensor outputs were then plotted against this ‘true’ value, and subsequent data adjustments were made using the derived equations.

In addition to personal exposure monitoring, participants were asked to complete a time-activity diary (see Supplementary Material A) to allow PM_2.5_ concentrations to be matched with the associated activity and microenvironment. The time-activity diary templates were structured in one-hour intervals, with participants providing a written description of their activities due to the diverse range of possibilities. Details about the microenvironment were gathered via checkboxes based on categories established by previous studies (e.g., [29]). These categories encompassed more general labels such as ‘transport’ and ‘public building’, as well as more specific settings within the home (e.g., ‘kitchen’, ‘bedroom’, ‘living room’). An ‘other’ checkbox was provided for instances where required.

Approximately 1-month post-baseline campaign and following a randomised control trial design, participants were split into two groups (control and intervention) in an approximate one-to-one ratio (Fig. 1). The control group (*n* = 13) conducted the second week of monitoring as they had the first, going about their usual day-to-day behaviours neither implementing co-developed nor prescribed behaviour changes. Intervention arm participants (*n* = 15) received the intervention (see Section “Intervention planning”).

At the end of each monitoring week, all participants completed a researcher administered MiniAsthma Quality of Life Questionnaire (mAQLQ; [30]). The mAQLQ is designed to measure various aspects of asthma-related health and wellbeing across four domains, namely physical symptoms, activity limitation, emotional function and environmental stimuli. It contains 15 questions and uses a seven-point scale with one indicating the most impairment and seven the least.

### Intervention planning

Data feedback and intervention planning conversations took place with fifteen participants via Zoom. These were structured around the ‘MAP of health behaviour change’, hereafter referred to as MAP, as detailed below.

#### Motivation

**Fig. 3:**
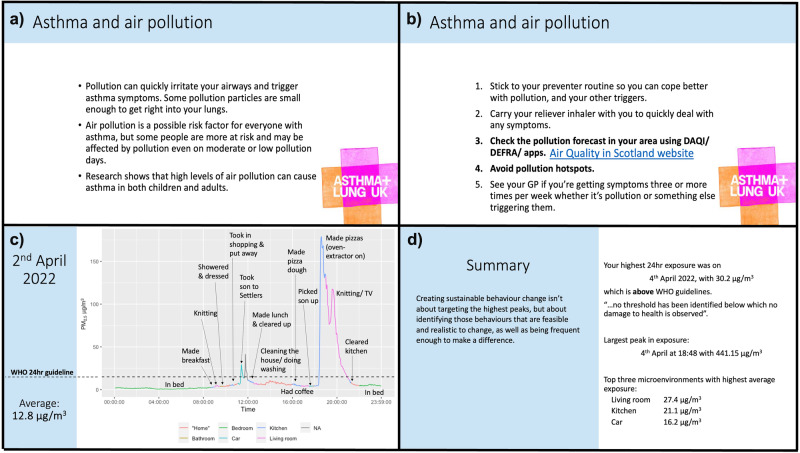
Slides shared with participants to target the motivation and action route to behaviour change. **a** and **b**) Bullet points outlining what is known about the links between asthma and air pollution (5.1 Information about health consequences; Michie et al. [31]) as detailed on the Asthma + Lung UK website in 2021 (9.1 Credible source; Michie et al. [31]). **c**) Personal exposure profile for one day of the baseline monitoring campaign. **d**) Summary slide.

To first target participants’ motivation to alter their behaviours to reduce their personal exposure, the intervention drew upon behaviour change techniques as defined within Michie et al.’s behaviour change taxonomy [31]. The taxonomy lists and describes 93 consensually agreed, distinct behaviour change techniques, and the intervention drew upon three; *5.1 Information about health consequences*, *9.1 Credible source* and *2.2 Feedback on behaviour* [31]. Using information readily available from Asthma + Lung UK (as a credible source), information about the health consequences was presented onscreen to each participant. This included information on air pollution as a potential asthma trigger, the links between air pollution exposure and asthma onset, acute asthma exacerbations as well as the impact of air pollution on the longer-term deterioration on respiratory health (Fig. 3a). In addition, an overview of Asthma + Lung UK’s recommended behaviour advice for managing asthma in relation to air pollution was presented to each participant (Fig. 3b). Following this, participants were presented with personalised exposure information from the previous monitoring week whereby the researcher guided the participant through the data highlighting peaks in exposure and the associated microenvironments and activities (taken from time-activity diary information), comparison with the WHO guideline for 24-hour exposure to PM_2.5_ and summarised average exposures across microenvironments (Fig. 3c, d). Regardless of study arm, if participants’ results indicated excessive exposure levels, we were ethically obligated to inform them and suggest exposure reduction strategies. Likewise, if participants’ diaries indicated that their asthma was poorly controlled based upon overreliance on their reliever inhaler, we would have recommended they contacted their healthcare professional. Since data review was a retrospective process rather than live, this intervention would have happened upon of completion of the monitoring campaign, however such interventions were not required.

#### Action

To target the action regulation route for behaviour change, participants and researchers co-developed the behaviour change intervention. This allowed the participant to plan (action and coping plans), implement, and self-regulate towards the intervention behaviour, with potential to be more effective in translating the intention into action [32]. These conversations were structured following the MAP template (Supplementary Material B) adapted from NHS educational materials and was shared onscreen and completed collaboratively. The role of the researcher was to facilitate this conversation and provide suggestions as needed, but the power and decision-making in choice of action was with the participant.

The outcome goal was to reduce personal exposure to PM_2.5_, however participants were able to add their own outcome goal(s) if desired. Participants then decided how they were going to achieve the outcome and set their behavioural goal reflecting on the air quality data feedback they had just received. This behaviour was then broken down in detail in the ‘action planning’ section of the template, with participants detailing when, where, how, the frequency and (if appropriate) with whom they would enact the behaviour change. Participants were then asked to identify barriers or challenges that could prevent them from successfully conducting the behaviour change before developing coping plans to help overcome these barriers. Behavioural changes were not specified or restricted to particular behaviours or microenvironments.

#### Prompts

The MAP planning conversation concluded with participants identifying the prompts and cues that could help them successfully enact the behaviour change. Since prompts and cues target the associative pathway (i.e., they don’t require deliberate thought or motivation to be necessary at the time of acting), this was participant-led. This would be vital for the development of sustainable behaviour change interventions based upon their own assessment of their personal context and the stimuli most likely to elicit their behavioural response.

### Analysis

#### Behaviour change interventions

Analysis was conducted on all co-developed interventions described in Section “Intervention planning” (*n* = 15; Fig. 1). The analysis focused on participants’ behavioural goals and the prompts they set to facilitate behaviour change. We employed Michie et al.‘s [31] behaviour change taxonomy to systematically code individuals’ main behaviour change interventions. This approach enabled us to thoroughly evaluate and classify the specific behaviour change techniques embedded within the co-developed interventions.

#### Personal exposure

Descriptive statistics were calculated for each participant’s PM_2.5_ baseline and follow-up personal exposure data across four different averaging periods; total exposure (the entire duration of the monitoring campaign), at-home exposure (exposure when the participants indicated they were within the home microenvironment), not-at-home exposure (exposure in any environment but the home), and intervention target behaviour (exposure during the enactment of the participant-chosen target behaviour, which the intervention was ultimately designed to address). Intervention target behaviours were identified from participants’ diary entries and coded as a binary variable based upon 2-min (raw) data. After co-designing the intervention, baseline data were revisited and activity targeted by the intervention coded. This variable was subsequently used to compare pre and post exposure for the intervention targeting behaviour change. Where pre-post data were available (*n* = 17 across the control and intervention arms; Fig. 1), average differences were calculated.

#### Self-reported health

Symptom occurrence for each hour was coded as a binary variable (symptoms experienced/ no symptoms experienced) and paired with hour-averaged exposure data. Asthma symptom prevalence was calculated as the percentage of hours within each individuals’ monitoring campaign with an experience of symptoms.

Since mAQLQ questions are equally weighted, participants’ mAQLQ scores were calculated using an individual’s mean score across the questions. Within-individual differences were calculated by subtracting the follow-up score from the baseline score and group medians calculated. Juniper [33] established that the Minimal Important Difference (MID), that is “the smallest difference in score which patients perceive as beneficial and would mandate, in the absence of troublesome side effects and excessive cost, a change in the patient’s management” ([34], pg. 408), is approximately 0.5. A score greater than 0.5 indicates a clinically meaningful improvement, less than −0.5 indicates a clinically meaningful deterioration, with values between considered clinically unchanged. However, when assessing the efficacy of an intervention across a group, such as in clinical trials, they suggest that simply comparing mean/median differences between treatment arms is not always suitable and does not account for the heterogeneity in responses. As such, an additional metric, the Number-Needed-to-Treat (NNT), was analysed to determine the number of patients who would need to receive the treatment for one individual to experience a clinically meaningful improvement in their asthma quality of life. This was calculated following the methodology proposed in Guyatt et al. [35] with tables used for these calculations included in Supplementary Material C.

## Results

### Participant characteristics

Of the 37 people enroled in the study, baseline data were collected for 28, with data excluded for nine, owing to ill health, sensor malfunction and diary-related issues (Fig. 1; [26]). Of the fifteen participants assigned to the intervention arm, all co-developed interventions. However, follow-up data were only collected/ analysed for nine, encountering similar issues as the baseline campaign. There was a similar data loss rate for the control arm whereby pre-post data were collected/analysed for eight of thirteen participants (Fig. 1).

Seventeen participants had pre-post exposure data available and were included in the final quantitative exposure analysis. Most participants were female (65%) and had an average age of 46.8 years (range: 24–74). Detailed demographic statistics for the sample as a whole and for the intervention arm participants who co-developed behaviour changes can be found in Supplementary Material D. The intervention group was representative of the overall study population.

### Tailored intervention behaviours

The predetermined outcome goal was to reduce personal exposure to PM_2.5_, though some participants chose to add an additional outcome goal (*n* = 6). These were pertaining to improved asthma symptoms (*n* = 2), the creation of new habits (*n* = 1), better asthma management (*n* = 2) and greater awareness of air pollution (*n* = 1).

All fifteen co-developed interventions were based within the home microenvironment (*n* = 15) and included largely positive action (e.g., “increasing ventilation” or “change cooking method”; *n* = 14). We identified three behaviour change techniques that participants drew upon as behavioural goals: *8.2 Behaviour substitution*; *12.1 Restructuring the physical environment* and *12.5 Adding objects to the environment*. The most frequent, *12.1 Restructuring the physical environment* (*n* = 10), included, for example, increasing or changing the current ventilation routine within the home. Three people set a behavioural goal of adding objects such as air purifiers or filters to a specific room within their home (*12.5 Adding objects to the environment*), with the remaining two substituting frequent cooking behaviours for alternative behaviours (e.g., opting to use a slow cooker instead of a gas stovetop; *8.2 Behaviour substitution*).

To support planned behaviour changes and to remind themselves to enact the intervention behaviour, participants drew upon three behaviour change techniques. Most frequently participants used prompts and cues as stimuli to remind them to enact the behaviour (*n* = 10; *7.1 Prompts and cues)*. Most frequently this manifested as visual prompts, such as placing stickers or sticky notes on or near to the object of interest (e.g., windows, extractor fans) to prompt the behaviour change (*n* = 8). This also included the use of alarms and phone alerts (as audio stimuli) as reminders to conduct the intervention behaviour (*n* = 2). Five participants used *7.8 Associative learning* which refers to the process of forming associations between a stimulus and a response. This included, for example, associating the action of starting to cook (specific stimulus) with turning on the extractor fan or opening a window (desired behaviour). Finally, two participants called upon reminders from co-habitees as a prompt to enact the behaviour (*3.1 Social support (unspecified)*).

### Impact of interventions on personal exposures

In McCarron et al. [26], we presented the week-long baseline PM_2.5_ data across all 28 participants. Here, we break this down for those in the control arm and intervention arm. At baseline, average exposure across the week for intervention arm participants was 10.9 μg/m^3^ (range: 2.7–26.2 μg/m^3^), which was higher than the average for control arm participants (7.5 μg/m^3^ (range: 1.0–21.8 μg/m^3^)). Intervention arm participants also had greater at-home personal exposure to PM_2.5_; their at-home exposure was 12.7 μg/m^3^ (17% higher than their baseline week-average), whereas control arm participants’ at-home exposure was 8.0 μg/m^3^ (6% higher than their baseline week-average).

Examining only the intervention-targeting behaviour (i.e., the behaviour participants identified in their action plans) for intervention arm participants (*n* = 9), average baseline personal exposure was 72.7 μg/m^3^ (range: 4.6–342.3 μg/m^3^). The average change across the intervention arm pre- and post-intervention was −43.9 µg/m^3^, ranging from −271.9 µg/m^3^ to −2.6 µg/m^3^. A reduction in personal exposure was observed across all participant intervention target behaviours (Table 1).

**Table 1:**
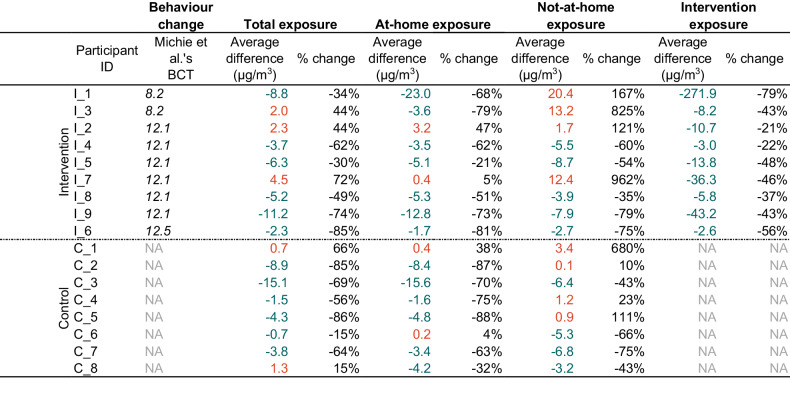
Participants’ change in personal exposures from the baseline week to the follow-up week.

Coded behavioural change techniques (BCT) (8.2 Behaviour substitution (e.g., using slow cooker rather than frying); 12.1 Restructuring the physical environment (e.g., opening windows); 12.5 Adding objects to the environment (e.g., adding an air purifier to a room)) are included for intervention arm participants.

Both the control and intervention arm reduced their at-home personal exposure to PM_2.5_ from baseline to follow-up campaigns. Within the home microenvironment, average difference in personal exposure was greater for intervention arm participants at −5.7μg/m^3^ (range: −23.0 to +3.2 μg/m^3^; Table 1) compared to the difference in at-home exposure for control arm participants of −4.7 μg/m^3^ (range: −15.6 to + 0.4 μg/m^3^; Table 1). The control arm experienced a change in average not-at-home exposure of −2.0 μg/m^3^ (range: −6.8 - +3.4 μg/m^3^; Table 1) between monitoring weeks. In contrast, the intervention arm saw an average change of +2.1μg/m^3^ (range: −7.9 to +20.4 μg/m^3^; Table 1) in not-at-home exposures between weeks. Examining differences in exposure across the two sampling weeks as a whole, the control arm had a greater change in average total exposure of −4.0 μg/m^3^ (ranging −15.1 to +1.3 μg/m^3^; Table 1). Comparatively, the intervention arm had a smaller average change of −3.2 μg/m^3^ (ranging −11.2 to +4.5 μg/m^3^; Table 1).

### Impact of interventions on self-reported asthma-related health

**Fig. 4:**
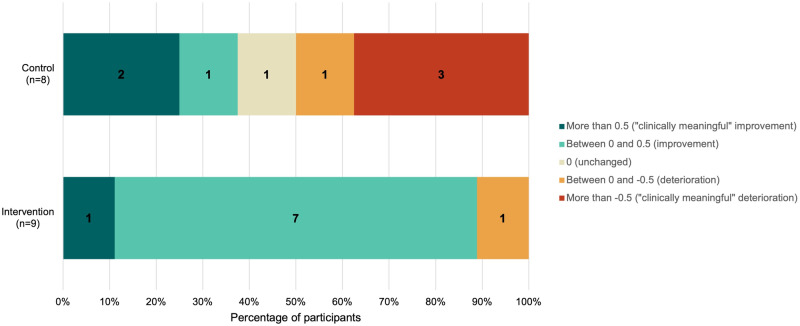
Participant individual differences in AQLQ score.

The greatest change in AQLQ scores was observed in the intervention arm, who had a change in their asthma quality of life score by a median of +0.3 compared to the control group’s change of −0.10 (Table 2). These scores, being within −0.5 and 0.5 (with a positive change indicating an improvement and negative change a deterioration) are not considered to be clinically significant for the groups overall [33]. For most intervention arm participants (*n* = 8), there was an improvement in AQLQ score, with one of the eight experiencing a clinically meaningful improvement (i.e., over 0.5). The control group experienced a smaller proportion of participants improving their scores (*n* = 3), and a greater proportion (*n* = 4) of participants experiencing a deterioration in their score (Fig. 4).

**Table 2:**
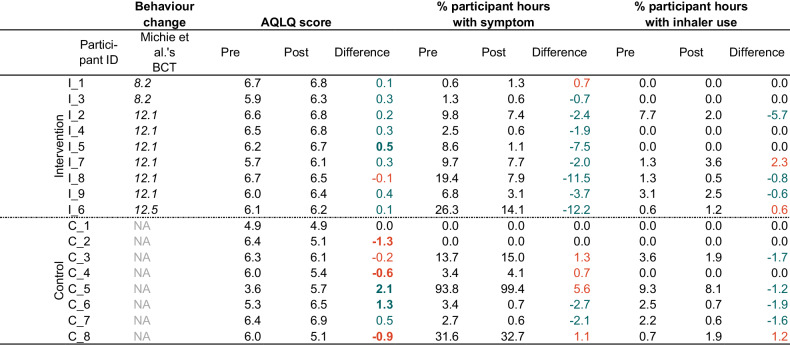
Participants’ change in self-reported asthma-related health outcomes from the baseline week to the follow-up week.

Coded behavioural change techniques (BCT) (8.2 Behaviour substitution (e.g., using slow cooker rather than frying); 12.1 Restructuring the physical environment (e.g., opening windows); 12.5 Adding objects to the environment (e.g., adding an air purifier to a room)) are included for intervention arm participants.

Examining change in the asthma quality of life domains, the greatest change in both groups was observed for symptoms, with a median improvement of 0.4 reported in the intervention group compared with a median deterioration of 0.3 in the control group. We observed no median change in activity limitation or environmental stimuli in the intervention group whereas a median improvement of 0.1 and 0.2, respectively, for the control group. The intervention group reported a median improvement of 0.3 for emotional function compared to no median change in the control group.

Based on the NNT, it was estimated that five patients would need to receive the intervention for one to experience a clinically meaningful improvement in asthma quality of life. In terms of resource efficiency, the symptoms domain can be most efficiently treated via this intervention, requiring three participants to receive treatment for one to experience a clinically meaningful improvement (Table 3).

**Table 3:** Number-needed-to-Treat (NNT) overall and across each of the AQLQ domains.

	Intervention mean difference mAQLQ score	Control mean difference mAQLQ score	Estimated proportion better on intervention	Estimated proportion better on control	Proportion benefitting from intervention	NNT
Overall	0.23	0.09	0.42	0.22	0.2	5.1
Symptoms	0.4	0.08	0.54	0.25	0.29	3.4
Activity limitation	0.03	0.19	0.38	0.25	0.13	7.7
Emotional function	0.22	0.04	0.42	0.28	0.14	7.1
Environmental stimuli	0.22	0.04	0.39	0.22	0.17	5.9

Asthma symptom prevalence, the percentage of hours with an asthma symptom recorded across the monitoring week, was examined. Between baseline and follow-up, the intervention arm reported an average difference of −4.6%, with all but one participant reporting a decrease in the proportion of time they reported an asthma symptom (Table 2). In comparison, control arm participants reported an average increase in symptom prevalence of 0.5%, with six of eight participants either experiencing no change or an increase in symptom prevalence (Table 2).

## Discussion

This pilot study has tested the viability of co-developing tailored interventions with people with asthma to reduce their personal exposure to PM_2.5_ and, subsequently, improve their self-reported asthma-related health. Using data feedback and structured intervention conversations following the NHS ‘MAP of health behaviour change’ [22] as the basis of intervention development, which to our knowledge has not previously been applied for reducing exposure to air pollution, we explore and discuss our findings below.

Personal exposure to air pollution is unique to an individual [36]. Though some factors that influence personal exposure are difficult - if not impossible - to control (e.g., where a person lives), personal exposure to air pollution can, to a degree, be modified by behaviour changes [13]. Recent research has emphasised the significance of personalisation of air quality data, suggesting that involving individuals in the process can enhance their engagement with air quality information [10]. Further, it has been suggested that personal exposure monitoring could be a useful step in the development of behaviour changes to support the management of cardiovascular and respiratory illnesses [26, 37]. We tested this in practice, and evidence from our pilot work with a small sample of participants shows that such an approach can work to firstly identify peaks in personal exposure and, secondly, target these using tailored behaviour change interventions to successfully reduce personal exposure.

Many studies have reported the ability of low-cost air quality monitors to effectively communicate personalised information and raise participant awareness of air quality, identify peaks in exposure and potential exacerbation risks (e.g., [37–40]). Consistent with prior research, our study has demonstrated that data feedback can effectively be used to identify specific activities or microenvironments where participants encounter elevated personal exposure levels. Notably, participants in our study directed their interventions towards behaviours that, at baseline, had exposure levels, on average, 17% higher than their average exposure across the baseline monitoring campaign. However, our study advances beyond the identification of exposure peaks; it has illustrated that individuals can translate their intentions into meaningful actions, finding that all intervention arm participants included in analyses reduced their personal exposure to PM_2.5_ whilst enacting the intervention behaviour. While Park et al. [41] report that personal exposure monitoring can modify attitudes, perceptions, and behavioural intentions, our research supports and demonstrates the efficacy of this approach to not only shape behavioural intentions but also to create effective targeted actions.

Though our results demonstrated efficacy on targeted personal exposures, our results yielded mixed results for participants’ at-home exposures. While, on average, the intervention arm experienced a greater reduction compared to the control arm, an increase in at-home exposure was observed for a small proportion of participants (two of nine) indicating non-universal impacts over longer durations. Furthermore, the control arm reduced their personal exposure to PM_2.5_ from baseline to follow-up campaigns (averaged across the week-long sampling period) to a greater degree than the intervention arm. This, in part, was influenced by an increase in the intervention arm’s not-at-home exposures (as generally uncontrollable microenvironments) between weeks, with the intervention group seeing an increase in exposure in these spaces (thus impacting the average overall exposure change). Considering that both groups reduced their at-home exposures between weeks (as controllable and more comparable environments), this suggests that personal exposure monitoring alone may enhance individuals’ awareness of their personal exposures, resulting in them, either consciously or subconsciously, altering their behaviours. Previously published work has shown the added value of personalised air quality data feedback over generic information (e.g., [42]), and taking into account the change in overall exposure (influenced by an increase in interventions arms not-at-home exposure), highlights the effectiveness of the at-home interventions further and the added value of data feedback and structured behaviour change planning on targeted and tailored exposure reduction. This demonstrates the ability of employing personal exposure monitoring and feedback, paired with structured behaviour change planning, as a method to identify peaks in personal exposure, reduce personal exposure and therefore, potentially, reduce the burden of air pollution on asthma symptom prevalence/control [26].

Asthma exacerbations caused by exposure to air pollution are a potentially preventable health risk [43]. Acute exposures are responsible for negative health consequences [44] since exposure to PM_2.5_ can induce an immediate physiological response characterised by inflammation of the airway, excess mucus secretion and tightening of the smooth muscle [5], resulting in common asthma symptoms such as wheeze and cough. Previous research on the same sample of participants as in this study has shown a positive association between acute PM_2.5_ personal exposure and symptom prevalence [26]. Therefore, reducing air pollution-related exposure events can yield immediate benefits for asthma-related health. Results from this study showed an average reduction in symptom prevalence within the intervention group (−4.6%), in contrast to the control group ( + 0.5%). Eight out of nine individuals in the intervention group reported experiencing fewer symptoms, while six out of eight in the control group reported no change or an increase in symptom prevalence. These findings, while for a small sample size, underscore the immediate impact of the intervention on health outcomes, supporting the use of personalised management strategies for asthma control [43, 45, 46].

Asthma symptoms are tangible indicators of an individual’s asthma control and overall health status [47]. However, solely focusing on clinical measures, such as peak expiratory flow (PEF), forced expiratory volume in one second (FEV_1_), or even symptom prevalence, overlooks the broader impact of the illness on overall wellbeing, which is an important component of asthma status in its own right [48]. Asthma quality of life offers a holistic measure of asthma-related health and wellbeing which can more clearly reflect the condition’s impact on a patient’s day-to-day life [49]. We hypothesised that reducing personal exposure to air pollution would result in improved AQLQ scores, reflecting better asthma control [50], increased activity capabilities, and improved emotional wellbeing [51] for individuals in the intervention group. Conversely, we expected scores in the control group to remain relatively stable. Our study revealed the most significant change in AQLQ scores occurred in the intervention group, with a median improvement of 0.3, compared to a median deterioration of 0.1 in the control group (Supplementary Material E). Consistent with symptom prevalence findings, the intervention arm improved their symptoms domain score by 0.40, while the control arm deteriorated by −0.30, providing evidence as to the potential health benefits provided by the intervention. This also supports that the implementation of individual-level interventions aimed at reducing the health effects of air pollution can lead to prompt and significant improvement in health [43]. Not only does this study point to the viability of intervention co-development for exposure reduction and improved asthma-related health, but our results indicate that, for symptom improvement in particular, this could be an efficient intervention. In comparison to other non-pharmacological asthma interventions such as practising mindfulness (e.g., 12; NNT = 7), the NNT for this intervention was comparatively more efficient, with five patients needed to treat for one to experience an overall improvement in asthma quality of life and three needed to treat for symptom improvement. The roll out of an intervention, co-developed between healthcare professionals and service users in a targeted manner (e.g., those unable to identify their triggers), utilising low-cost sensor technology and established behaviour change tools, could, therefore be a feasible solution to improve asthma management and control. This approach could also reduce healthcare utilisation in a cost-effective manner, with prevention being favourable over treatment [52].

While symptom prevalence and environmental stimuli can be objectively measured, activity limitation and emotional function are more nuanced and subjective. These domains rely more on individuals’ self-perceptions, emotional states, and personal interpretations of how asthma affects their daily lives. This subjectivity forms a crucial and novel element in our approach and the essence of co-developing tailored interventions for individual-level behaviour change. There have been several arguments made against individual-level behaviour changes to reduce exposure versus emission reduction strategies, such as the burden of responsibility they place on the individual and their potential to widen existing disparities [11]. However, findings from this study suggest that individual-level interventions can be empowering for susceptible groups, enabling them to regain control over their exposure and health while maximising personal choices [53]. This is evident in our findings, as the intervention arm experienced no median change in activity limitation indicating the implementation of an individual-level intervention as no more burdensome than inaction. Further, the median improvement in emotional function (with these questions within the mAQLQ pertaining to feelings of frustration, feeling afraid and feeling concerned) suggested that co-developed interventions may offer broader benefits beyond exposure reduction and improved symptom prevalence, but also work to the lessen feelings of anxiety surrounding their asthma and empower them to reduce their personal exposures [54]. Stanescu et al. [55] report that anxiety in individuals with asthma is frequently linked with activity limitation and a perceived lack of control over their capabilities. This perception of control has been recognised as a key factor associated with quality of life [56] by instilling individuals’ confidence in managing their condition [57]. Consequently, Adams [57] argues that placing greater emphasis on perceived control appears justified as a central aspect of asthma management. As a means of improving overall quality of life for individuals with asthma, the co-development of behaviour change interventions based on data feedback provides them with an additional tool for taking charge of their health and mitigating their exposure to air pollution.

Control, in addition to lessening feelings of vulnerability, is a fundamental component in the development of coping strategies aimed at reducing people with asthma’s exposure to air pollution [58]. Perceived lack of control, on the other hand, can hinder the development of behaviour change [10] and has been found as a main factor in non-adherence to the behavioural advice communicated as part of top-down air quality communications, for example, from the UK’s DAQI or Canada’s Air Quality Health Index [59, 60]. Generally, people do not have control over their wider outdoor environment; they cannot (majorly) influence ambient air quality, in most cases they cannot avoid leaving their home to go to work and, for some, they cannot avoid physical activity outdoors (e.g., walking to work or school). Yet the behavioural advice communicated as part of the dissemination of air quality information is focused on avoidance behaviours in the outdoor environment. Though previous studies have found that people with asthma, owing to greater awareness of their personal vulnerability, are more likely to engage in avoidance behaviour [61, 62], this is not consistent with our findings. Individuals have little control [11], and little perceived behavioural control [25] in the outdoor environment, evidenced by no participants developing behaviour change interventions for the outdoor microenvironment. Rather than participants co-developing avoidance behaviours when faced with the ability to choose the behaviour change to implement, participants opted for positive (i.e., “increasing ventilation”) actions within the home, an obvious contrast with more traditional reduction and avoidance advice (e.g., “remain indoors and keep activity levels low”, “reduce physical exertion, particularly outdoors…”). Ultimately, participants chose to change behaviours that they felt they could control, increasing their sense of self-efficacy. Thus, reframing how air quality related behavioural advice is communicated, putting more emphasis on the behaviours or environments where people feel that they have control, and framing these as more positive actions [63], could be a more effective strategy for the sustained uptake of protective actions and reduce the burden of air pollution-related asthma exacerbations.

Effective and sustainable behaviour change interventions require tailoring to both reflective and automatic processes [32]. Reflective processes are deliberate and require thought, consideration and cognitive effort to perform the intended behaviour action whereas automatic processes are non-conscious, instead prompted or cued by environmental, social, cognitive or psychological stimuli which signal an automatic associated behavioural response [23]. Participants self-implemented their behaviour change by opting for visual or audio prompts in their environment or social stimuli to remind them to take action, targeting behaviour change via the automatic and reflective pathway [64], which, since the automatic process is less cognitively demanding, could be beneficial for sustainable behaviour change. Additionally, participants choice of visual and audio prompts signifies an adaptive response to air pollution as a largely imperceptible problem [65]. This emphasises the critical role of data feedback to highlight exposure to air pollution in the home which previously would have been unperceivable [25] and highlights the potential of this approach to co-develop sustainable behaviour change interventions.

## Limitations and recommendations for future work

Owing to the nature of a pilot study, this study was not powered to assess the differences in personal exposure or health measures between study arms. Our findings have demonstrated the viability of this method for exposure identification and effective intervention co-development for reduced personal exposure to PM_2.5_ and improved self-reported asthma-related health. This paper creates an opportunity for future work to adopt this method and apply it to a larger sample size for more robust analysis.

The small sample in this study is due, at least in part, to preventable data loss for reasons such as illegible or incomplete diaries. We recommend that future studies should adopt alternative means of diary collection, for example in a digital format (e.g., [66]). Additionally, while we made efforts to recruit a generally representative sample, reliance on voluntary participation introduces a potential for selection bias, with it likely individuals who are more concerned or affected by air pollution more likely to volunteer, and those with greater resources, such as time and energy, may find it easier to participate.

Collecting subjective data from participants can pose significant challenges. In this study, although it was designed as a randomised controlled trial, it was conducted in a non-blinded manner, meaning that participants were aware of their assigned study arm. This awareness raises the possibility that the perceived benefits of the intervention may have been overstated in the intervention arm while understated in the control arm. For instance, holding other variables constant, not receiving the intervention should not have adversely affected control arm participants. However, we observed a decrease in AQLQ scores and an increase in symptom prevalence in this group. These findings may suggest reporting bias, given the inherently subjective nature of AQLQ responses, which rely on participant recall and self-assessment, especially in the context of a non-blinded study. For future studies and applications of this method, we recommend incorporating more objective health measures, such as spirometry tests [67, 68] and, where possible, conducting this in a blinded manner.

It should also be noted that ambient air quality in Scotland, which influences personal exposure, is generally much better compared to other countries. Conducting similar research in countries or cities with higher levels of ambient air pollution would be beneficial.

Finally, future studies should consider different asthma phenotypes. Considering the array of phenotypes, for some individuals, air pollution will simply not be an asthma trigger and symptomology and clinical features will differ between individuals. Focussing on a particular phenotypic subgroup, or more broadly patients with poorly controlled asthma, presenting frequently at their GP or A&E department and who are unsure of their triggers, may be more beneficial, insightful and cost-effective. Additionally, exploring the application of the method to different respiratory conditions such as COPD and bronchiectasis would also be worthwhile.

## Conclusions

This study set out to test the ability of data feedback and structured intervention co-development to create tailored behaviour changes and reduce individual exposure to PM_2.5_ and improve self-reported asthma-related health. We have demonstrated that: (1) personalised data feedback can help individuals with asthma to identify peaks in their personal exposure to air pollution; (2) these can be targeted with co-developed behaviour change interventions; (3) co-developed interventions can reduce personal exposure to PM_2.5_ during the targeted behaviour; and (4) co-developed interventions can improve self-reported asthma-related health. These pilot findings demonstrate that such an approach warrants further feasibility testing with a larger group of participants. Further feasibility testing should also test this approach for other respiratory conditions potentially exacerbated by air pollution, for example, COPD and bronchiectasis.

As well as demonstrating the efficacy of the co-developed interventions, we have shown that this is potentially an efficient approach (based upon NNT), which, if applied in a targeted manner (i.e., with patients with poorly controlled asthma), could represent a high-value and low-cost intervention. As such there is potential to integrate aspects of the approach into existing practices, such as asthma review appointments in healthcare settings, however this would need further testing around feasibility, acceptability and cost-effectiveness.

While this study focused on individual-level behaviour changes, this needs to be considered within the context of the suite of measures needed to reduce air pollution exposures encompassing top-down policies and bottom-up behaviour changes, such as explored in this study. This intervention gives those most vulnerable to the health effects of air pollution exposure an additional ‘tool’, allowing them to take control over their personal exposure to air pollution and help them to improve their asthma-related health.

## Supplementary information

Supplementary Material A

Supplementary Material B

Supplementary Material C

Supplementary Material D

Supplementary Material E

reproducibility-and-quality-checklist

## Data Availability

Data will be made available upon request.
