# Supplementary material for: Piloting co-developed behaviour change interventions to reduce exposure to air pollution and improve self-reported asthma-related health
Source: J Expo Sci Environ Epidemiol. 2024 Apr 12;35(2):242–53. doi: 10.1038/s41370-024-00661-2 (PMC12009737; doi:10.1038/s41370-024-00661-2)

Supplementary Material B – Behaviour change planning template completed collaboratively with participants based upon the ‘MAP of health behaviour change’ (22).


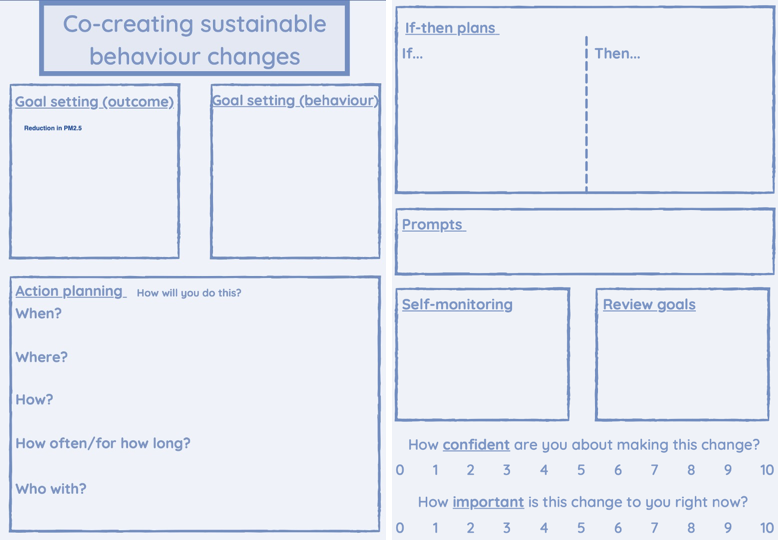

Supplement: Supplementary file 2 — Supplementary Material B [file 41370_2024_661_MOESM2_ESM.docx]
