# Supplementary material for: Piloting co-developed behaviour change interventions to reduce exposure to air pollution and improve self-reported asthma-related health
Source: J Expo Sci Environ Epidemiol. 2024 Apr 12;35(2):242–53. doi: 10.1038/s41370-024-00661-2 (PMC12009737; doi:10.1038/s41370-024-00661-2)
Supplement: Supplementary file 3 — Supplementary Material C [file 41370_2024_661_MOESM3_ESM.docx]

Supplementary Material C – Demographic tables for a) all participants with pre-post exposure and health data and b) for intervention arm participants who co-developed a behaviour change intervention (adapted from McCarron et al. 2023).

1. **All with pre-post (n=17)**

| **Participant characteristic** | **Control** | **Intervention** |
| --- | --- | --- |
| **Age (years, 𝑥 ̅ (range))** | 50.5 (24-70) | 43.6 (24-74) |
| **Sex (n (%))** |  |  |
| Female | 5 (29.4) | 6 (35.3) |
| Male | 3 (17.6) | 3 (17.6) |
| **Other respiratory condition (n (%))** |  |  |
| No | 7 (41.2) | 9 (52.9) |
| Yes | 1 (5.9) | 0 (0.0) |
| **Pregnant (n (%))** |  |  |
| No | 8 (47.1) | 9 (52.9) |
| Yes | 0 (0.0) | 0 (0.0) |
| **SIMD Quintile (n (%))** |  |  |
| 1 | 0 (0.0) | 0 (0.0) |
| 2 | 0 (0.0) | 1 (5.9) |
| 3 | 4 (23.5) | 3 (17.6) |
| 4 | 0 (0.0) | 3 (17.6) |
| 5 | 4 (23.5) | 2 (11.8) |
| **Type of dwelling (n (%))** |  |  |
| Apartment | 3 (17.6) | 3 (17.6) |
| Semi-detached house | 2 (11.8) | 1 (5.9) |
| Detached house | 2 (11.8) | 2 (11.8) |
| Semi-detached bungalow | 0 (0.0) | 0 (0.0) |
| Detached bungalow | 1 (5.9) | 1 (5.9) |
| Detached cottage | 0 (0.0) | 1 (5.9) |
| Terraced house | 0 (0.0) | 1 (5.9) |
| **Number of residents (𝑥 ̅, (range))** | 2 (1-3) | 2.6 (2-5) |
| **Live with pets (n (%))** |  |  |
| No | 4 (23.5) | 3 (17.6) |
| Yes | 4 (23.5) | 6 (35.3) |
| **Live with smoker (n (%))** |  |  |
| No | 8 (47.1) | 9 (52.9) |
| Yes | 0 (0.0) | 0 (0.0) |
| **Have a solid fuel burner (n (%))** |  |  |
| No | 7 (41.2) | 6 (35.3) |
| Yes | 1 (5.9) | 3 (17.6) |
| **Type of stovetop (n (%))** |  |  |
| Gas | 3 (17.6) | 4 (23.5) |
| Other (electric, induction) | 5 (29.4) | 5 (29.4) |
| **Urban-rural Classification (n (%))** |  |  |
| Accessible rural | 0 (0.0) | 2 (11.8) |
| Accessible small town | 0 (0.0) | 1 (5.9) |
| Large urban | 2 (11.8) | 1 (5.9) |
| Other urban | 5 (29.4) | 3 (17.6) |
| Remote small town | 1 (5.9) | 0 (0.0) |
| Very remote rural | 0 (0.0) | 1 (5.9) |
| Very remote small town | 0 (0.0) | 1 (5.9) |

1. **Intervention (n=15)**

| Participant characteristic | Statistic |
| --- | --- |
| Age (years, 𝑥 ̅ (range)) | 43.7 (24-74) |
| Sex (n (%)) |  |
| Female | 10 (66.7) |
| Male | 5 (33.3) |
| Other respiratory condition (n (%)) |  |
| No | 15 (100.0) |
| Yes | 0 (0.0) |
| Pregnant (n (%)) |  |
| No | 15 (100.0) |
| Yes | 0 (0.0) |
| SIMD Quintile (n (%)) |  |
| 1 | 0 (0.0) |
| 2 | 3 (20.0) |
| 3 | 4 (26.7) |
| 4 | 4 (26.7) |
| 5 | 4 (26.7) |
| Type of dwelling (n (%)) |  |
| Apartment | 6 (40.0) |
| Semi-detached house | 3 (20.0) |
| Detached house | 2 (13.3) |
| Semi-detached bungalow | 0 (0.0) |
| Detached bungalow | 2 (13.3) |
| Detached cottage | 1 (6.7) |
| Terraced house | 1 (6.7) |
| Number of residents (𝑥 ̅, (range)) | 2.7 (1-5) |
| Live with pets (n (%)) |  |
| No | 5 (33.3) |
| Yes | 10 (66.7) |
| Live with smoker (n (%)) |  |
| No | 15 (100.0) |
| Yes | 0 (0.0) |
| Have a solid fuel burner (n (%)) |  |
| No | 12 (80.0) |
| Yes | 3 (20.0) |
| Type of stovetop (n (%)) |  |
| Gas | 5 (33.3) |
| Other (electric, induction) | 8 (53.3) |
| Missing | 2 (13.3) |
| Urban-rural Classification (n (%)) |  |
| Accessible rural | 3 (20.0) |
| Accessible small town | 2 (13.3) |
| Large urban | 3 (20.0) |
| Other urban | 4 (26.7) |
| Remote small town | 1 (6.7) |
| Very remote rural | 1 (6.7) |
| Very remote small town | 1 (6.7) |
