# Supplementary material for: Piloting co-developed behaviour change interventions to reduce exposure to air pollution and improve self-reported asthma-related health
Source: J Expo Sci Environ Epidemiol. 2024 Apr 12;35(2):242–53. doi: 10.1038/s41370-024-00661-2 (PMC12009737; doi:10.1038/s41370-024-00661-2)
Supplement: Supplementary file 4 — Supplementary Material D [file 41370_2024_661_MOESM4_ESM.docx]

Supplementary Material D – Number-needed-to-treat tables calculated based on the calculated proportion of patients who benefited from receiving treatment (33).

| **Overall** | **NNT** | **5.1** |  |
| --- | --- | --- | --- |
|  | **Treatment** | | |
| **Control** | Improved (0.11) | Unchanged (0.89) | Deteriorated (0.00) |
| Improved (0.25) | 0.03 | 0.22 | 0.00 |
| Unchanged (0.38) | 0.04 | 0.33 | 0.00 |
| Deteriorated (0.38) | 0.04 | 0.33 | 0.00 |
|  |  |  |  |
| **Symptoms** | **NNT** | **3.5** |  |
|  | **Treatment** | | |
| **Control** | Improved (0.33) | Unchanged (0.67) | Deteriorated (0.00) |
| Improved (0.38) | 0.13 | 0.25 | 0.00 |
| Unchanged (0.13) | 0.04 | 0.09 | 0.00 |
| Deteriorated (0.50) | 0.17 | 0.34 | 0.00 |
|  |  |  |  |
| **Activity limitation** | **NNT** | **7.7** |  |
|  | **Treatment** | | |
| **Control** | Improved (0.00) | Unchanged (1.00) | Deteriorated (0.00) |
| Improved (0.25) | 0.00 | 0.25 | 0.00 |
| Unchanged (0.38) | 0.00 | 0.38 | 0.00 |
| Deteriorated (0.38) | 0.00 | 0.38 | 0.00 |
|  |  |  |  |
| **Emotional function** | **NNT** | **7.2** |  |
|  | **Treatment** | | |
| **Control** | Improved (0.33) | Unchanged (0.56) | Deteriorated (0.11) |
| Improved (0.38) | 0.13 | 0.21 | 0.04 |
| Unchanged (0.25) | 0.08 | 0.14 | 0.03 |
| Deteriorated (0.38) | 0.13 | 0.21 | 0.04 |
|  |  |  |  |
| **Environmental stimuli** | **NNT** | **6.1** |  |
|  | **Treatment** | | |
| **Control** | Improved (0.33) | Unchanged (0.56) | Deteriorated (0.11) |
| Improved (0.25) | 0.08 | 0.14 | 0.03 |
| Unchanged (0.50) | 0.17 | 0.28 | 0.06 |
| Deteriorated (0.25) | 0.08 | 0.14 | 0.03 |
