# Supplementary material for: Piloting co-developed behaviour change interventions to reduce exposure to air pollution and improve self-reported asthma-related health
Source: J Expo Sci Environ Epidemiol. 2024 Apr 12;35(2):242–53. doi: 10.1038/s41370-024-00661-2 (PMC12009737; doi:10.1038/s41370-024-00661-2)
Supplement: Supplementary file 5 — Supplementary Material E [file 41370_2024_661_MOESM5_ESM.docx]

Supplementary Material E – AQLQ tables

| Intervention | **Total Difference** | **0.07** | **0.40** | **0.20** | **0.33** | **0.07** | **0.27** | **0.33** | **-0.13** | **0.53** |
| --- | --- | --- | --- | --- | --- | --- | --- | --- | --- | --- |
|  |  |  |  |  |  |  |  |  |  |  |
|  | Symptoms | -0.20 | 0.80 | 0.40 | 0.60 | 0.40 | 0.40 | 0.20 | -0.20 | 1.20 |
|  | Activity limitation | 0.00 | 0.00 | 0.00 | 0.25 | 0.25 | 0.00 | -0.50 | 0.25 | 0.00 |
|  | Emotional function | 0.00 | 0.00 | 0.33 | 0.67 | -0.67 | 0.67 | 0.67 | 0.00 | 0.33 |
|  | Environmental stimuli | 0.67 | 0.67 | 0.00 | -0.33 | 0.00 | 0.00 | 1.33 | -0.67 | 0.33 |

| Control | **Total Difference** | **2.07** | **0.00** | **1.27** | **-0.93** | **-1.33** | **-0.20** | **-0.60** | **0.47** |
| --- | --- | --- | --- | --- | --- | --- | --- | --- | --- |
|  |  |  |  |  |  |  |  |  |  |
|  | Symptoms | 2.00 | -0.60 | 2.00 | -1.80 | -0.60 | 0.00 | -1.60 | 1.20 |
|  | Activity limitation | 2.50 | 0.00 | 1.75 | -0.75 | -1.75 | 0.25 | -0.75 | 0.25 |
|  | Emotional function | 2.33 | 0.67 | 0.67 | -1.00 | -1.67 | -0.67 | 0.00 | 0.00 |
|  | Environmental stimuli | 1.33 | 0.33 | 0.00 | 0.33 | -1.67 | -0.67 | 0.67 | 0.00 |
